# Patient motivators of postoperative electronic patient-reported outcome symptom monitoring use in thoracic surgery patients: a qualitative study

**DOI:** 10.1186/s41687-024-00766-0

**Published:** 2024-07-25

**Authors:** Meghan C. O’Leary, Elizabeth Kwong, Chase Cox, Amanda L. Gentry, Angela M. Stover, Maihan B. Vu, Jessica Carda-Auten, Jennifer Leeman, Gita N. Mody

**Affiliations:** 1 Lineberger Comprehensive Cancer Center, The University of North Carolina at Chapel Hill, Chapel Hill, NC USA; 2 Gillings School of Global Public Health, The University of North Carolina at Chapel Hill, Chapel Hill, NC USA; 3 Carolina Health Informatics Program, The University of North Carolina at Chapel Hill, Chapel Hill, NC USA; 4 Department of Surgery, The University of North Carolina at Chapel Hill, Chapel Hill, NC USA; 5 School of Medicine, The University of North Carolina at Chapel Hill, Chapel Hill, NC USA; 6 Center for Health Promotion and Disease Prevention, The University of North Carolina at Chapel Hill, Chapel Hill, NC USA; 7 School of Nursing, The University of North Carolina at Chapel Hill, Chapel Hill, NC USA

**Keywords:** Patient-Reported Outcomes, Thoracic surgery, Postoperative, Symptom monitoring, Qualitative research

## Abstract

**Background:**

Electronic patient-reported outcome (ePRO) systems can be used to engage patients in remote symptom monitoring to support postoperative care. We interviewed thoracic surgery patients with ePRO experience to identify factors that influenced use of ePROs to report their symptoms post-discharge.

**Method:**

This qualitative study used semi-structured telephone interviews with adults who underwent major thoracic surgery at an academic medical center in North Carolina. Individuals who enrolled in symptom monitoring, completed at least one ePRO survey, and were reachable by phone for the interview were included. The ePRO surveys assessed 10 symptoms, including validated Patient-Reported Outcome Common Terminology Criteria for Adverse Events (PRO-CTCAE) measures and thoracic surgery-specific questions. Surveys, offered via web-based and automated telephone options, were administered for four weeks post-discharge with alerts sent to clinicians for concerning symptoms. The interviews were guided by the Capability, Opportunity, Motivation model for behavior change (COM-B) and examined factors that influenced patients’ completion of ePRO surveys post-discharge. Team members independently coded interviews and identified themes, informed by COM-B. We report descriptive statistics (demographics, number of surveys completed) and themes organized by COM-B components.

**Results:**

Of 28 patients invited, 25 (89%) completed interviews from July to October 2022. Participants were a median 58 years, 56% female, 80% White, and 56% had a history of malignancy. They completed 131/150 (87%) possible ePRO surveys. For capability, participants reported building ePROs into their routine and having the skills and knowledge, but lacking physical and emotional energy, to complete ePROs. For opportunity, participants identified the ease and convenience of accessing ePROs and providers’ validation of ePROs. Motivators were perceived benefits of a deepening connection to their clinical team, improved symptom management for themselves and others, and self-reflection about their recovery. Factors limiting motivation included lack of clarity about the purpose of ePROs and a disconnect between symptom items and individual recovery experience.

**Conclusions:**

Patients described being motivated to complete ePROs when reinforced by clinicians and considered ePROs as valuable to their post-discharge experience. Future work should enhance ePRO patient education, improve provider alerts and communications about ePROs, and integrate options to capture patients’ complex health journeys.

**Supplementary Information:**

The online version contains supplementary material available at 10.1186/s41687-024-00766-0.

## Abstract (fr)

People who undergo thoracic surgery often experience pain and other symptoms while recovering at home. These symptoms can be severe and may reduce overall quality of life and potentially result in some patients returning to the hospital for future treatment. Electronic Patient-Reported Outcomes can be used as a method for having patients regularly track and report any symptoms they experience while at home, and how severe those symptoms are, using digital technology such as an online survey or automated phone survey. Surgical care team members may then follow up with patients about their symptoms. More information was needed about the patient experience with completing these surveys about their symptoms. In this study, we interviewed patients who had completed Electronic Patient-Reported Outcomes after thoracic surgery to understand what may (or may not) have impelled them to participate and to learn how to improve the use of these surveys for patients. This study found that patients generally felt they were able to complete the symptom surveys. Key motivators included feeling more connected to their surgeon by completing the symptom surveys and having the opportunity to reflect on how their recovery was going at home. However, patients also discussed not having a clear understanding of the purpose of the symptom surveys and how their responses might affect their care. The study findings highlight the need for improved patient education and indicate that improvements to the survey questions and to how surgeons review patients’ responses may be needed.

## Background

Thoracic surgery, which is performed approximately 530,000 times annually in the United States, is associated with high symptom burden that may persist beyond the acute postoperative recovery period, including pain, fatigue, and dyspnea [1–6]. For example, an estimated one-third of patients experience chronic pain following minimally-invasive thoracic surgery [5], and approximately 60% of patients report dyspnea following lung cancer resection, with persistently more severe dyspnea one year post-surgery [6–7]. Failure to promptly manage symptoms during the postoperative period may lead to diminished quality of life or increased readmission rates [2, 8].

Digital health technologies, such as electronic patient-reported outcome (ePRO) systems, can be used to engage patients in remote symptom monitoring to support their postoperative care [10]. Extensive prior research has generated a set of valid and reliable PRO measures [11–14]. Earlier research has demonstrated the clinical utility of ePRO systems [15–16] and the potential for improved data quality with ePROs compared to paper and pen [17]. Documented benefits of ePRO symptom monitoring include symptom control, reduced complications, fewer emergency room visits and hospitalizations, and improved health-related quality of life among diverse surgical and cancer patient populations [9, 16, 18–21]. Additional advantages include improved patient-provider communication and increased patient agency in their recovery process [15].

In a previous study on the impact of ePRO monitoring on quality of life (“Thoracic Surgery Patient-Reported Outcomes,” [*TSPRO 1.0*, NCT04342260]), we enrolled thoracic surgery patients in postoperative symptom monitoring via a web-based ePRO platform and qualitatively assessed a subset of patients’ barriers and facilitators to survey completion [22]. Facilitators included the ease of using the platform and increased awareness of their own symptoms. Barriers to ePRO use included lack of email access, poor physical and mental health particularly in the early days after returning home, and lack of clarity about the value of ePRO use within clinical care.

In the current study (*TSPRO 2.0; NCT05311670*) [23], we implemented four types of design changes to address these barriers to completing ePROs. First, while surveys were previously administered via a web-based platform alone, we added an automated telephone option (interactive voice response or IVR) to minimize digital technology and connectivity gaps [14]. Second, we revised the survey schedule to be less frequent, changing from daily surveys to twice a week, to minimize patient burden and nonadherence in a period characterized by high pain and symptoms. Third, we reduced the number of survey items from 15 to 10 to minimize burden and eliminate symptoms with low prevalence. Lastly, we disseminated brochures to patients about why ePROs are collected.

Here, we build on our prior work and use interviews with patients to learn about their experiences with our design changes. Guided by the Capability, Opportunity, and Motivation Model for Behavior Change (COM-B) [24], we explored the factors influencing their initial uptake of and ongoing engagement with ePRO symptom monitoring.

## Methods

The parent study, *TSPRO 2.0*, evaluated the implementation of ePROs postoperatively as part of research tied to routine care delivery. In this study, we qualitatively assessed patient motivators of ePRO use in the context of a large academic public hospital in North Carolina. The University of North Carolina at Chapel Hill (UNC) Institutional Review Board approved these studies.

### Inclusion/exclusion criteria

Inclusion criteria were having recently undergone a major thoracic surgery including chest wall incisions and overnight admission and being 18 years or older, able to understand and speak English, and able to complete web-based or telephonic symptom surveys postoperatively. Exclusion criteria were being pregnant or currently incarcerated, planning to undergo foregut surgery, and being unable to provide informed consent due to dementia, altered mental status, or a psychiatric condition. Recruitment occurred in-person in the postoperative wards or clinic. For this qualitative analysis, we added the inclusion criteria of completing at least one (out of six) postoperative ePROs and being reachable by phone.

### ePRO symptom monitoring

**Fig. 1:**
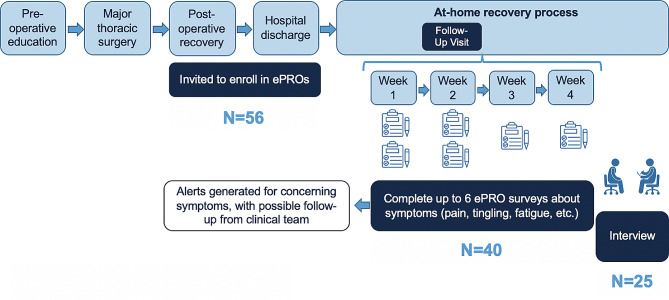
Schematic of recruitment and study activities for *TSPRO 2.0* parent study and qualitative sub-study. We report the number of individual patients who participated in each of these activities. Fifty-six patients were invited to participate in remote symptom monitoring via ePRO symptom surveys in our *TSPRO 2.0* parent study. Of the 56 invited patients, 40 consented to participate in *TSPRO 2.0*. Of the 40 enrolled patients, 25 completed an individual interview about their experience with ePRO symptom monitoring.

Figure 1 summarizes study participation for the parent study and patient interviews. Forty (74%) of 56 eligible patients who were invited to participate enrolled in the parent study and were administered six ePRO symptom surveys over a four-week period during recovery, twice weekly for two weeks and then once weekly for two weeks. We selected this schedule to capture symptoms more frequently during the first two weeks post-discharge, while reducing the overall number of surveys in response to patient feedback on survey burden.

Participants had two options for completing ePROs: (1) an existing web-based platform accessed via computer or smartphone or (2) IVR (automated telephone calls). Automated reminders were sent via email to those who selected the web-based option and by scheduled, automated phone call for those who selected IVR. A study coordinator followed up with patients who missed two surveys.

Each ePRO symptom survey contained 10 items assessing the frequency and severity of symptoms associated with thoracic surgery including pain, shortness of breath, and cough, along with an option to write-in additional concerns. Most items were selected from validated and reliable National Institutes of Health (NIH) Patient-Reported Outcome Common Terminology Criteria for Adverse Events (PRO-CTCAE) measures [11–14]. To tailor symptom monitoring to our population, we included a few items specific to post-surgical care such as redness or discharge at the incision site. We collaborated with UNC’s Patient-Reported Outcomes Core (PRO Core), which provides scientific support with administering and managing ePROs within cancer research, to program the selected symptom items into their PRO Core system [25]. While ePROs were not integrated into the electronic health records (EHR), alerts were generated if the patient reported severe or worsening symptoms, or utilized the free-text field, and sent electronically to the patient’s surgical team via the EHR. The surgical team was directed to review and contact the patient as needed per routine clinical workflow.

### Interviews

Interview participants were considered if they had completed at least one of six ePROs. Because there was relatively high adherence, we defined low adherence as completing less than 80% of offered surveys, and ensured representation of patients with low adherence among those interviewed. Interviews were conducted until thematic saturation was achieved.

The study team contacted participants by phone to invite them to participate in an individual interview about their symptom monitoring experience. Interviews, which lasted approximately 30–45 min, were conducted by phone to improve ease for patients who were in the postoperative recovery period and, thus, potentially still experiencing high symptom burden. We used semi-structured interviews to gain an in-depth understanding of factors that influenced patients’ engagement with ePRO symptom monitoring from when they were approached about symptom monitoring through completion of ePROs. Qualitative researchers (MBV, JC) from the UNC Connected Health for Applications & Interventions (CHAI) Core conducted interviews. Participants received a $25 gift card for their time.

### Frameworks

The interview guide (Supplemental File 1) was informed by the COM-B framework [24]. Questions focused on patients’ experiences completing the symptom surveys (which we considered a type of behavior change), including whether they had the knowledge and abilities to complete ePROs (capability), what factors supported ePRO completion (opportunity), and what impelled them to complete (or not complete) ePROs (motivation). Additionally, we had an emerging theme code for topics that were most important to patients.

**Fig. 2:**
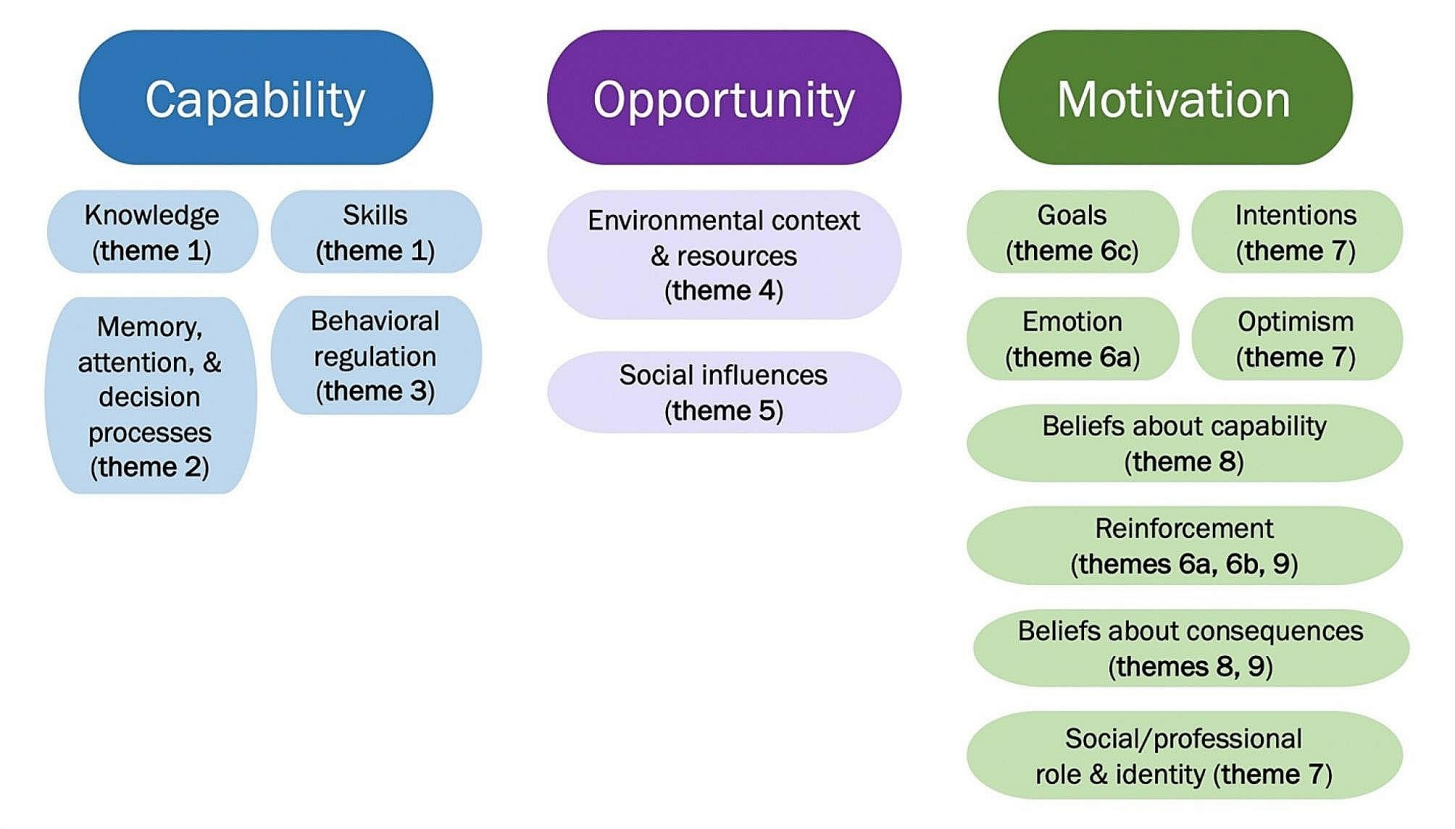
Integration of COM-B Components and TDF Domains. This figure shows how our identified themes (see Table 2) map onto the COM-B components and TDF domains.

In the analysis phase, we integrated the Theoretical Domains Framework (TDF) [26], a framework for understanding barriers and facilitators to behavior change that has previously been used with COM-B [27–29]. We added this framework to establish continuity in how we defined the COM-B components (Fig. 2).

### Qualitative analysis

Interviews were audio-recorded, transcribed by a professional transcription service, and de-identified. We (MCO, MBV, JC, JL, GNM) co-developed an initial codebook based on the interview questions and data collection notes, and pilot-tested it by independently coding a subset of transcripts. During this iterative process, we fine-tuned concept definitions, revised decision rules, and ensured replicability across coders. We (MCO, MBV, JC) then applied the final codebook (Supplemental File 2) to the remaining transcripts. Coding was performed using the software Dedoose. Standard consensus coding guidelines [30, 31] were followed, where any emerging theme or discrepancy was captured and reconciled through discussion. We (MCO, MBV, JC) reviewed individual Dedoose-generated code reports and, for each code, prepared a narrative description of the identified themes and subthemes and included illustrative quotes. Finally, using our combined COM-B/TDF framework, we (MCO, GNM, JL) further refined and organized themes and subthemes by COM-B component.

### Quantitative analysis

We report descriptive statistics including patient demographics (age, sex, race/ethnicity), surgical history (surgery type, history of malignancy), postoperative events (complications or readmissions within 30 days), and ePRO completion (modality, number of symptom surveys completed). Demographic and clinical variables were determined using EHR data with clinical outcomes reviewed by the surgeon on our team. Median and interquartile ranges are reported where applicable.

## Results

### Demographic and clinical characteristics

Table 1 summarizes the characteristics of the 25 thoracic surgery patients who completed interviews. Participants had a median age of 59 (interquartile range: 20.5). The cohort was comprised of 56% females, 80% non-Hispanic White individuals, and 12% non-Hispanic Black individuals. More than half (56%) had a history of lung cancer or another type of malignancy. More participants had completed minimally invasive surgery (60%) than open surgery (40%). Twenty (80%) participants completed ePROs via the web-based platform and 5 (20%) via IVR. Of the 150 possible surveys offered, 131 (87%) were completed across participants, a median of 6 surveys per interviewee (interquartile range: 1).

**Table 1:** Characteristics of interview participants

Characteristic	*N* (%)
Overall	25 (100%)
Sex	
Female Male	14 (56%) 11 (44%)
Age	
Median (interquartile range)	59 (20.5)
Age category	
<40 40–49 50–59 60–69 70–79	3 (12%) 4 (16%) 7 (28%) 5 (20%) 6 (24%)
Race/ethnicity	
Non-Hispanic White Non-Hispanic Black Non-Hispanic Asian	20 (80%) 3 (12%) 2 (8%)
Type of thoracic surgery	
Open Minimally invasive	10 (40%) 15 (60%)
History of lung cancer or another malignancy	
Yes No	14 (56%) 11 (44%)
Complication at 30 days after thoracic surgery	
Yes No	10 (40%) 15 (60%)
Readmission at 30 days after thoracic surgery	
Yes No	1 (4%) 24 (96%)
Modality used to complete ePROs	
Interactive voice recording (IVR) Web-based platform	5 (20%) 20 (80%)
ePRO symptom survey completion status (6 max)	
Low (0–2 surveys) Moderate (3–4 surveys) High (5–6 surveys) Median (Interquartile range)	2 (8%) 2 (8%) 21 (84%) 6 (1)

### Interview themes

Nine themes were identified related to the capability, opportunity, and motivation to engage in symptom monitoring postoperatively. Table 2 provides additional quotes by theme. Figure 2 illustrates how each theme aligns with the COM-B components and corresponding TDF domains.

**Table 2:** Themes related to the capability, opportunity, and motivation for ePRO symptom monitoring among postoperative thoracic surgery patients (*n* = 25)

Theme	Example quotes
**Capability—do patients have the ability to complete ePRO symptom surveys?**	
1. Participants have the knowledge and skills to complete ePROs	“They were straightforward, I didn’t have any problem with ’em.” —Patient 10 “It’s just multiple choice, very easy. It’s focused on my symptoms, my particular situation, so no challenges completing the survey itself.” —Patient 20 “All the questions were just basic questions, and I thought everything was great, so I didn’t have any problems filling it out at all.” —Patient 9
2. Completing ePROs can require emotional and physical energy	“Then, too, it was a constant reminder of what I’ve been through…I had my moments where I felt depressed. I was tired of being cut on. I went through all those emotions while being in the hospital…to have to keep reliving what I’ve been through was torture at times as well.” —Patient 17 “Well, not difficult at all except that I was in a whole lotta pain.” —Patient 8 “At the time I didn’t feel like it was always a priority for me to do that because I had increased pain issues post-op, so my goal was to focus on my own physical symptoms and managing those day-to-day, not completing the questionnaire.” —Patient 14
3. Participants were able to build ePRO completion into their daily routine	“I accepted it as part of my routine, so there wasn’t any issue with doing it. It wasn’t a burden for me.” —Patient 15 “It wasn’t that hard because I was still in the healing process. I pretty much really had all the time to kill.” —Patient 18 “I might’ve checked my email once or twice just to see if I had ’em, but I check it pretty regularly anyway. I think, one time, they sent me a reminder email ’cause I was a day late on the survey, but I generally check my email every two to three days, so it was never really an issue.” —Patient 11 “I check my inboxes pretty frequently, and then I tend to—I tend to solve whatever comes in immediately, so it wasn’t an issue for me.” —Patient 7
**Opportunity—do external factors facilitate ePRO symptom survey completion?**	
4. ePRO interface and structure facilitated completion of ePROs	“When I could do it at my pace, it was great. That’s the reason I had mine online… You even had a little grace period to get ’em done. When I was havin’ a good moment, I would fill out that survey. Convenience was a great thing with that.” —Patient 21 “It, honestly, wasn’t too hard. It wasn’t too bad, ‘cause there weren’t a ton of questions. It didn’t take too long to fill out.” —Patient 13 “It was easy to answer. It didn’t take very long.” —Patient 22 “It was easier for me because I can use a computer, but sometime, when I’m usin’ it, I get most of my stuff wrong, incorrect…the phone was easier, like talkin’ to you.’" —Patient 19 “I know, though, occasionally I got the email that said,'Hey, a new survey’s available to you,' and, whether it was the same day or the next day, I got the reminder if I hadn’t yet taken it. Then I would take it.” —Patient 8 “The second one was similar, so I thought,'Oh, I’ve already done that one.' Then they gave it to me a second time. [Laughs] Well, they sent the first one a second time, and I don’t know why, but the second one they sent, and they said,'Hey, you haven’t done this one.'” —Patient 2
5. The involvement of providers helped to legitimize the importance of ePROs	“When they approached me while I was in the hospital, and they said that Dr. [Name] was involved—I don’t know whether [they were] involved in it, but [they] wanted to know the response to the questionnaire. That was my understanding. The way I felt about [them] and the way I felt comfortable with [them and their] whole staff, I felt comfortable to go ahead and do the questionnaire.” —Patient 16 “Well, it was important. Dr. [Name] asked if I could do it, and I would do anything for [them].” —Patient 24
**Motivation—are patients motivated to complete ePRO symptom surveys?**	
6. Participants were motivated to complete ePROs by their perceived individual benefits: A. Accompaniment and a deepening connection with their care team (i.e., feel less alone) B. Care improvement (e.g., addressing complications) C. Self-reflection (i.e., setting expectations, tracking progress)	**A. Accompaniment and a deepening connection with their care team (i.e., feel less alone)** “There was almost a way of having them not necessarily there, but since they couldn’t be there in person, it was like having my medical team still asking, is this okay? Is that okay? Are you doing this? Are you doing that? Having that continuation gave me a sense of security.” —Patient 3 “‘Cause a lotta times you get in your mind that okay, I’m really just a number and a insurance claim. When they take time out—like I said, I’ve been through this going on 10 years now. I have never had a doctor call me afterwards and ask me,'Well, how do you feel about this?' and try to give me information to help me along with my recovery process. That was definitely a great thing.” —Patient 17 “Even though I wasn’t there, they were still monitoring me through...the questions I had answered. I wasn’t alone. They were still there for me to take care of any—to answer any questions of the different symptoms I had, any...problems I had with recovery. I felt that they were always there at my fingertips to help me.” —Patient 24 “Showed me that y’all was interested in my health and wellbein’ even after it was over with.” —Patient 19 “I don’t know if it makes too much sense, but it was nice having somebody, not calm me down off the ledge, ’cause I wasn’t really freaking out, but just having somebody there, just some sort of edification that I’m doing all right” —Patient 11 “The past experiences I’ve had where I went to other hospitals, you just go home, and you don’t hear anything from anybody, and to be asked how you’re doin’, that’s kind of nice.” —Patient 9 “I did because I did get those callbacks, but I wasn’t expecting the callbacks, so that was a nice surprise. I didn’t get one after every single survey, I don’t think, but I did get a few. It was a nice surprise that they would call if there was somethin’ on there that they wanted more information about.” —Patient 8 **B. Care improvement (e.g., addressing complications)** “The minute [Nurse] called me… concerned about the responses that I had provided in terms of just still being short of breath and not feeling like I was healing the way I should have been based on my previous experiences, I knew it was worth it to do.” —Patient 20 “What I did like that happened a couple times, depending on the answers I put down—somebody from the thoracic team did call me to follow up on my symptoms. I thought that was very beneficial.” —Patient 13 “I spoke with [Nurse name] a couple of times…I think I had written something about the pain in two of the surveys, so [they] called back then I think to see if I was coping with it and to give me some advice on what else to do to get through it” —Patient 9 “Actually every time I completed one...in the comments part I would write,'I don’t like the way the incisions look.' Somebody would call me the next day…in fact, one time, this is amazing, I was in the hospital for the EMG test, and I got a call from [Name] in Dr. [Name]'s service that they had gotten that, that I didn’t like the incision looked. They got me in in 30 minutes, so I didn’t have to leave the hospital. I just went to the other clinic and got it with them.” —Patient 6 “I did expect ’em to call me ’cause I was still havin’ a lot of pain in a spot that I didn’t think was s’pose to have pain” —Patient 1 **C. Self-reflection (i.e., setting expectations, tracking progress)** “I think it helped me to think about my situation at that point—how I was feeling. Gives a little more perspective, rather than just—I have a tendency to be a little stoic sometimes and ignore things. So it gave me an opportunity to think about exactly how I was feeling.” —Patient 12 “…if you’re answering questions about it and maybe you answer it one way and then the next week you answer it a different way, you might—things aren’t necessarily improving. It might throw off a red flag one way or another. I take it just like a weekly, I guess, check-in on yourself and how your progress is going.” —Patient 13 “...newer patients like myself, like I said, we were going into the unknown. We don’t know how to feel. Are we supposed to do this? Are we supposed to feel like this? It just helps, for lack of a better word, the newer patients cope with what they’re feeling.” —Patient 24 “Like I say, just helping keep me honest to look out for symptoms and me being aware.” —Patient 11 “Well, I feel like that I would’ve been more educated. I could’ve got more educated on how I’m feelin’ or how I was feelin’, or you educatin’ someone else, how they would feel if they went through that surgery, what to expect” —Patient 4
7. Participants were motivated to improve care	“Just because you do things, sometimes, we, as people, get in a rut. We do things the same way every single time, but there’s always time for change that makes things a little bit better. Even when what we do works, change can make it work better, sometimes.” —Patient 21 “I think they should if it’s doctors, surgeons, and nurses, and also the pharmacists better understanding of care. Because I had to take specific medicines because I’m allergic to some pain medicine. Even the medicines I was given, we had to dilute it some. I think they could [get] a better understanding and take mental notes so they know how to go through it next time with someone else. Just be aware that, hey, this is a possibility.” —Patient 18
8. The simplicity of the ePROs limited their fit to individual experiences	“… the questionnaire was almost a little too simple. It’s not that I had anything more to say, but when I saw it and that it gave me the choices for did you experience fever, and it says, not at all, and gives you the gradation questions, after that I thought, wonder if I had a fever…this seems like a single question to cover what could be a more complex situation.” —Patient 3 “I think some of the answers that choices could have been a little more fine-tuned…I had to just sort of pick the best one, but it wasn’t necessarily the best one for me.” —Patient 12 “Maybe rewording it differently or different questions, I’m not really sure which way to go. It wasn’t bad, the same questions, ’cause it did give you a idea of your progression…I just didn’t realize they were gonna be the same.” —Patient 24 “After surgery I’m still gonna always be short of breath because I have been since I was sick. They said it’s just gonna be a thing because it’s like body memory. That’s the only thing your body knows. It’s not urgent where I’m desperately out of breath. It’s not an emergency. That’s where I felt like it really wasn’t that important. Because I was telling them it wasn’t anything to stress and worry about. I guess they didn’t take note of that. Then I got a phone call after being asked,'Oh, are you okay? Do you need to be rushed in the ER?' I’m like,'No, I’m fine. I was just answering questions.'” —Patient 18
9. Patients lacked clarity on the purpose of ePROs and how their responses would be used	“I’m not sure I even knew from what their description was what I would be doing and how it would help…What could they have done differently? I just think it’s that education and really what to expect, and I just don’t think that was crystal clear.” —Patient 14 “It wasn’t for me. It was for other people who might be going through or gettin’ ready to go through or whatever.” —Patient 5 “I didn’t know all those surveys I was filling out for five or six weeks, I didn’t know those were like real time and being looked at by the office. I thought it was just data collection. I would have been more detailed and provided more information and maybe not have clicked through them as quickly if I knew that somebody was really monitoring that and would act based on what I was putting in there.” —Patient 23 “I have no idea what they’re doin’ with this information. Probably just assessing…recuperation time and pain levels, et cetera.” —Patient 11 “Just that it could affect you as your care ’cause it did affect mine. Like, I wasn’t expecting a call from the surgical team when I put that in, and they did it twice, so the very next day. They had to have been seeing it somehow, which I didn’t think they were seeing it.” —Patient 6 “Like I say, I was on a lot of meds, and I don’t know whether… I can’t remember no one talkin’. I can’t. I can’t say that they didn’t call or they did. I just can’t really remember.” —Patient 4

### Capability

Participants overwhelmingly described having the knowledge and skills to complete the surveys (theme 1). Describing the surveys as “straightforward” and “easy” to complete, participants generally reported being able to understand the symptom questions and how to respond. One participant explained, “*It’s just multiple choice*,* very easy. It’s focused on my symptoms*,* my particular situation*,* so no challenges completing the survey itself*” (Patient 20). Participants reported knowing how to access new surveys, and remembering to complete them on their own or with the help of automated reminders. Most participants recalled receiving at least one reminder about the surveys and indicated that the reminder did typically nudge them to complete the survey. Seven participants reported that they either did not receive a reminder, likely because they promptly completed each survey, or found them unnecessary because they could remember on their own or did not want additional emails.

While participants largely agreed they were capable of completing surveys, a few shared that survey completion required emotional and physical energy (theme 2). This included the emotional toll of being reminded about their surgical experience. One participant said completing ePROs “*was a constant reminder of what I’ve been through…I had my moments where I felt depressed…I went through all those emotions while being in the hospital…to have to keep reliving what I’ve been through was torture at times*” (Patient 17). Completing ePROs also required patients to expend time and energy that could otherwise be focused on recovery. “*I had increased pain issues post-op*,* so my goal was to focus on my own physical symptoms and managing those day-to-day*,* not completing the questionnaire*” (Patient 14). Although this theme was mentioned by a small subset of participants, it was salient to those who reported needing to prioritize between completing the surveys and focusing on their recovery in other ways.

Participants reported being able to build ePRO symptom monitoring into their regular routines (theme 3). One participant explained, “*I accepted it as part of my routine*,* so there wasn’t any issue with doing it. It wasn’t a burden for me*” (Patient 15). This routinization of symptom monitoring included integrating the surveys into morning routines or regular screen time.

### Opportunity

With respect to the social and environmental factors that facilitated survey completion, participants acknowledged the ease of the ePRO interface and format (theme 4). Participants pointed to the multiple-choice format of the survey items, low number of survey questions, and consistency of questions across surveys as facilitators. Several participants stated that this structure allowed each survey to be completed quickly (usually 5 minutes or less). Web-based survey users noted the convenience of accessing the survey via the online platform when they were ready. One participant explained, “*When I was havin’ a good moment*,* I would fill out that survey. Convenience was a great thing with that*” (Patient 21). However, one participant experienced some local connectivity issues, and another experienced a challenge distinguishing between new versus previously completed surveys. IVR users reported no connectivity issues. One explained: “*[IVR] was easier for me because I can use a computer*,* but sometime*,* when I’m usin’ it*,* I get most of my stuff wrong*,* incorrect…the phone was easier*” (Patient 19).

In terms of social influences, participants described their providers’ involvement as helping to legitimize the importance of ePRO completion (theme 5). This included any type of acknowledgement of symptom monitoring by a member of the clinical team. Even when participants were unclear on how their surgeon and other clinicians were directly involved with ePROs, having an understanding that they were supportive of ePROs made participants feel comfortable completing the surveys. “*The way I felt about [my doctor] and the way I felt comfortable with [them and their] whole staff*,* I felt comfortable to…do the questionnaire*” (Patient 16). In this way, ePROs were viewed as connected to routine care because they were endorsed in some way by providers.

### Motivatio

Participants described three ways that they benefited from ePRO monitoring and that motivated them to complete symptom surveys (theme 6). First, many participants were motivated by a deepening connection with their surgical team that they experienced by engaging in ePROs (theme 6a). They described feeling less alone and “being seen” by their providers after receiving follow-ups to their survey responses. One patient said, “*Even though I wasn’t there*,* they were still monitoring me through whatever I had answered…I felt that they were always there at my fingertips to help me*” (Patient 24). Having a way to experience the care they previously received in the hospital in their own homes motivated patients. “*…it was like having my medical team still asking*,* is this okay? Is that okay? Are you doing this? Are you doing that? Having that continuation gave me a sense of security*” (Patient 3). This sense of accompaniment was identified as a strong motivator for ongoing ePRO participation among those who received a direct follow-up.

Second, several participants provided specific examples of how symptom monitoring improved their care—whether by addressing a complication or managing a particular symptom (theme 6b). For example, one participant shared that a member of their clinical team located them in the hospital waiting for an unrelated appointment to inspect their incision, which they had reported some concerns about via a symptom survey. Other participants recalled talking through a particular symptom, such as pain or shortness of breath, with a clinician by phone in response to their survey. “*What I did like that happened a couple times*,* depending on the answers I put down—somebody from the thoracic team did call me to follow up on my symptoms*” (Patient 13). Participants described these calls as an opportunity to elaborate on and receive support with their symptom burden.

Third, participants reported being motivated to engage in the self-reflection afforded by symptom monitoring (theme 6c). Completing the surveys at multiple time points allowed patients to track their recovery progress over time and have dedicated time to reflect on how their symptoms were changing. One participant noted that symptom monitoring was “*just helping keep me honest to look out for symptoms and me being aware*” (Patient 11). Participants described setting expectations for recovery based on the symptoms included in ePROs. The surveys acted as a type of checklist for common symptoms to watch out for during recovery and a way to self-assess whether their symptoms were improving or declining.

In addition to the individual benefit of ePROs, participants were motivated by the potential to improve care for thoracic surgery patients more broadly (theme 7). Some participants described wanting to ensure that their providers understood the full health journeys that patients experience at home during recovery. One participant explained, “*I just think it’s important for a doctor or whoever to know what you go through when you go home and what symptoms you’re having and whatnot*,* so they can better understand*” (Patient 25). Others described being motivated to complete the surveys so that their providers could use the information learned about symptom trajectories and complications to improve treatment and better set expectations for other patients. Even outside of the research context, participants viewed symptom monitoring as an opportunity to contribute to healthcare innovation to improve surgical care for all patients.

Participants also reported factors that made them less motivated to complete symptom monitoring. Some participants found that the survey items failed to capture their individual experiences (theme 8). “*I think some of the answers that choices could have been a little more fine-tuned…I had to just sort of pick the best one*,* but it wasn’t necessarily the best one for me*.” (Patient 12). This was due, in part, to limited response options for each survey item (e.g., 4-point Likert scales ranking symptom severity) or inclusion of symptoms that participants had not experienced during recovery. Another participant noted that the ePROs generated follow-ups from the clinical team for symptoms they did not perceive to be of concern. Participants also perceived less value in the surveys for addressing more complex health experiences.

Motivation was also hindered when participants were unclear about the purpose of completing ePROs and how their responses would be used (theme 9). Multiple participants reported that they did not know whether or how their clinical team might use the information reported in their surveys. Some expected their responses to be used for research purposes only and were unaware of their connection to clinical care. A participant explained, “*I would have been more detailed and provided more information and maybe not have clicked through them as quickly if I knew that somebody was really monitoring that and would act based on what I was putting in there*” (Patient 23). A patient who completed only one of the surveys described difficulty in understanding and remembering the objective: “*I can’t remember no one talkin’…I can’t say that they didn’t call or they did. I just can’t really remember*” (Patient 4). Regardless of level of ePRO adherence, participants commonly reported a need for improved education about the purpose of remote symptom monitoring upfront to improve motivation.

## Discussion

In this qualitative analysis, we found that thoracic surgery patients generally felt they had the capability, opportunity, and motivation to complete ePROs post-discharge. Participants appreciated being more connected to the surgical team while recovering at home, the attention to their concerning symptoms (whether through self-reflection or clinician follow-up), and found the process of completing ePRO symptom surveys to be relatively simple. Challenges affecting capability and motivation for some patients to complete ePROs included the added effort during a difficult recovery process, lack of fit of the symptom items assessed, and uncertainty about the intent.

Participants who were contacted by a provider following an alert overwhelmingly identified this as a positive experience that fostered their relationship with the care team and provided them with clinical benefit. This experience reinforced their motivation to complete subsequent ePROs because they had seen the value of ePROs to their care experience. Notably, while the benefits of PRO-based care, such as helping to improve patient-provider consultations during future visits, have been documented in other settings [32–33], studies have also shown that some patients felt a disconnect from the health system when they received no response or a superficial response [34] and concern that they would be forgotten in the digital system [35]. In our study, patients who did not receive a clinician follow-up, likely because they did not have alert-generating symptoms, did not report this type of disconnect. However, they commonly reported not knowing if or how their responses were reviewed or used by their clinical team. Improved education is needed upfront to set clearer expectations about how ePRO survey responses may be used, by whom, and under which circumstances they may receive a follow-up contact from their providers. Design changes like providing feedback to patients that their ePROs have been reviewed may also be considered.

This study was designed to iteratively assess design changes informed by our prior qualitative inquiries in ePRO patient participants. Adding the IVR modality appeared to reduce a previously reported connectivity barrier, as those who selected this option did not report issues accessing the surveys and lack of technology access was no longer a reported reason for ineligibility to the parent study. Excluding survey items shown to be uncommon for generating alerts among thoracic surgery ePRO participants seemed to reduce reports of redundancy in the ePRO questions. However, despite reducing the survey frequency and length, participants continued to report a barrier of diminished energy during the postoperative recovery process.

This analysis raises some important considerations regarding scalability of postoperative ePROs. For example, we found that reminders were an important facilitator of ePRO completion, echoing prior research [36–37]. Most participants were responsive to ePRO reminders, but some required a follow-up call from the study coordinator and implementing these direct, personalized reminders may be difficult on a larger scale. We think it will be important to use automated responses to increase adherence as a first attempt and identify who on the clinical team might be tasked with direct reminders. Patient navigators may be uniquely positioned for this activity, as ePROs are one way to get a better sense of how patients are doing and personalize their care [38–39]. For patients who are non-responsive to ePROs, navigator calls may provide a critical touchpoint.

More consideration is needed regarding the level of integration of ePROs within our health system’s EHR. We used an external system for ePROs requiring program staff to ensure alerts were sent to clinicians. This approach is potentially less efficient as we work to scale up the use of ePROs within clinical care. However, it allows for increased customization of the ePROs and reports, which is important as we learned in this study the need for an adaptive ePRO process that best fits individuals’ unique recovery experiences. To address these types of tradeoffs, the Symptom Management Implementation of Patient-Reported Outcomes in Oncology (SIMPRO) consortium summarized the advantages and disadvantages of freestanding ePRO systems, external systems that interface with the EHR, or fully integrated ePRO/EHR systems [40]. We are currently exploring avenues with respect to the level of integration of ePROs within our EHR, recognizing that there is still uncertainty in the preferred approach.

Patients’ feedback on their experiences completing ePROs suggests opportunities to improve remote symptom monitoring. Despite introducing brochures intended to improve patients’ understanding of the purpose of ePROs, participants discussed their lack of clarity about the use of ePROs in routine care. This finding aligns with prior research [34] highlighting uncertainty or misunderstanding among patients regarding how information collected with ePROs will be used outside of a research context and identifies a need for improved educational materials such as provider scripts about the intervention and its connection to their clinical care [41]. Patient education could also be built into the ePRO itself to reinforce the purpose of completing the surveys at each touchpoint with the patient.

Enhanced provider education about ePROs and communications about symptom monitoring with patients may also be needed. Consistent with prior research [35], participants reported that any type of validation of the importance of ePROs from their clinical team, even if a small acknowledgement, increased their willingness and motivation to participate. Despite these endorsements, there remained uncertainty about whether their providers would use the collected symptom information. Clinical teams implementing ePROs may wish to use PROmunication strategies [42], a theory-driven manual for guiding dialogue about PROs, for example, to improve patient understanding and engage patients in conversations around their reported symptoms. Offering ongoing provider trainings may help providers to learn, practice, and remember to use the recommended language in interactions with patients [43].

Another recommendation is to explore ways to allow for more tailored symptom content while still maintaining the simplicity of the symptom survey format. The content of the standardized survey items did not always reflect patients’ individual health journeys, for example if they experienced different types of complications than those assessed, if the answer options were not nuanced enough to describe the burden, or if the symptoms were relevant but chronic and not in need of acute management. There may be a disconnect between what patients consider to be important or burdensome symptoms and what providers perceive to be most important or alert-worthy [44]. These are areas for future research.

It is important to consider how our study design may have affected findings. Interviews occurred a median of 22 days after participants’ last completed survey, with all participants completing the interview within three months. This relatively recent recall time likely allowed patients to more easily remember and reflect on their ePRO experiences; however, recall bias may have been an issue for some patients, particularly postoperatively. Additionally, it is possible that our use of a theoretical framework to inform our interview guide and analysis may have unintentionally prioritized certain aspects of patients’ experiences. However, we wanted to understand the combination of factors that influence uptake and ongoing completion of surveys by including questions relevant to COM-B. To mitigate potential biases, we provided opportunities throughout the interviews for patients to address aspects of the ePRO experience that were salient to them and ways to improve their experiences. We included emergent codes and conducted interviews until no new topics were generated, and we integrated the TDF into our analysis to be intentional about carefully considering multiple aspects of each COM-B component.

This study has limitations. Despite our efforts to recruit a diverse mix of participants, those interviewed are not representative of the demographic diversity of the health system’s patient population. Nonresponse bias was likely an issue, as patients who initially declined to participate in ePROs were not included. It will be important to consider their perspectives as these patients may experience additional barriers to ePRO participation (e.g., more health complications, etc.). Additionally, most participants were highly adherent with the intended ePRO survey schedule and, thus, may have been more willing to complete an interview than those who were less motivated or faced more challenges completing ePROs. Lastly, it was challenging at times to disentangle findings specific to being involved in a research study versus use of ePROs as part of routine care, but we attempted to prioritize the latter during our coding process.

## Conclusions

As the implementation of ePROs grows as part of postoperative care, we found promising results that thoracic surgery patients with postoperative symptom monitoring experience largely reported having the capabilities, opportunities, and motivators to complete ePROs. We identified several patient-informed recommendations that may help to improve the implementation and effectiveness of ePROs within routine care.

### Electronic supplementary material

Below is the link to the electronic supplementary material.

Supplementary Material 1

## Data Availability

The datasets used and analyzed during the current study are available from the corresponding author upon reasonable request.

## Abbreviations

CHAI: Connected Health for Applications & Interventions
COM-B: Capability, Opportunity, Motivation model for behavior change
EHR: Electronic health record
ePRO: Electronic patient-reported outcome
IVR: Interactive voice response
NIH: National Institutes of Health
PRO: Patient-Reported Outcomes
PRO-CTCAE: Patient-Reported Outcome Common Terminology Criteria for Adverse Events
SIMPRO: Symptom Management Implementation of Patient-Reported Outcomes in Oncology
TDF: Theoretical Domains Framework
UNC: University of North Carolina at Chapel Hill
