# Supplementary material for: Patient motivators of postoperative electronic patient-reported outcome symptom monitoring use in thoracic surgery patients: a qualitative study
Source: J Patient Rep Outcomes. 2024 Jul 25;8:81. doi: 10.1186/s41687-024-00766-0 (PMC11282008; doi:10.1186/s41687-024-00766-0)
Supplement: Supplementary file 1 — Supplementary Material 1 [file 41687_2024_766_MOESM1_ESM.docx]

Supplemental File A. Interview Guide

**Section 1: Establishing a Context for the Discussion to Follow**

1. Tell me about what problems led to your recent surgery.
   1. What type of surgery did you have?
   2. What kind of symptoms were you experiencing before surgery? Physical symptoms? Emotional well-being?

**Section 2: Symptom Questionnaire Usage and Impressions**

*I’d like to learn more about your experience with the post-surgery symptom questionnaire.*

1. Do you remember completing questionnaires about your symptoms after surgery?
   1. Approximately how many symptom questionnaires have you completed to date?
2. Who asked you to complete the assessment?
3. Have you been completing the post-surgery symptom questionnaire by phone or via the Internet?
   1. What were the reason(s) for you choosing the phone? choosing the Internet?
   2. What challenges if any have you experienced when completing the questionnaire?
   3. How easy or difficult have you found it to remember to complete the questionnaire? Why?
4. What kind of reminders have you received to complete the questionnaire?
   1. How helpful have those reminders been?
   2. What kind of reminders would you like to receive to help you remember/prioritize completing the symptom questionnaire?
5. How did you fit completing the questionnaire into your day to day activities while recovering?
6. How important was it to you to complete the symptom questionnaire?
   1. What made it seem important (or unimportant)?
   2. What could your healthcare team do or say that would further convince you of the questionnaire’s importance?
7. In what ways, if at all, do you feel like you are benefiting from completing the questionnaires?
8. To what extent do you think completing the questionnaires could help you?

**Section 3: ePRO Symptom Questionnaire Response Utility**

1. What is your understanding of how your doctor or nurse plan to use/used the information from your questionnaires?
2. How interested would you be to review your symptom questionnaires with your providers?
   1. If yes: Why are you interested in reviewing your symptoms with your provider?
   2. If no or unsure: Why do you feel this way?
3. What is your level of interest in seeing the information you reported on the questionnaire?
   1. When would you like to see the information you reported?
   2. How interested would you be to compare your responses to those of other patients like you?
   3. Do you use MyChart?
      1. If Yes: How do you typically use MyChart?
      2. If Yes: How do you feel about the information you provided on the questionnaire being available on MyChart?
      3. If No: Continue with next question.

**Section 4: Feasibility of symptom questionnaires for patients**

1. Who, if anyone, has helped you fill out the questionnaire?
   1. Can you tell me a little more about how they helped you?
2. Overall, how easy or difficult has it been to report your symptoms right after getting home from the hospital (in the first one or two weeks)? What can be done to improve this?

**Section 5. Closing**

1. Would you recommend that other post-surgery patients fill out this kind of symptom questionnaire to help their surgeons and nurses take better care of them? Why/why not?
2. We’ve talked about many important things today. Is there anything else you would like us to know or suggestions for improving the study?

Supplemental File B. Codebook

| **Code Concept** | **Code Label** | **Code Definition** |
| --- | --- | --- |
| **Section 1. Context and Background** | | |
| What led to recent chest surgery and what type of surgery patients underwent. Also, who approached patient to be part of the research study. | **Symptoms &**  **Surgery Type** | Use this code when participants comment on the symptoms (physical or emotional) they experienced leading up to their recent thoracic surgery and the type of surgery they underwent.  Also use this code when participants discuss who approached and asked them to complete the symptom management questionnaires. |
| **Section 2. ePRO Symptom Questionnaire Patient Impressions** | | |
| Phone or online reception to ePROs and their reasons for choosing that modality | **ePRO Modality**  **(Opportunity; Environmental context & resources)** | Use this code when participants comment on whether they chose to use the phone (IVR telephonic recording) or online to complete their ePRO questionnaires and their **reasons for choosing their respective modality**.  NOTE: Patients did not have the option to switch from IVR to online (or vice versa) during the study. If they said they started by web and then completed by phone, they actually chose to complete the ePRO with the study coordinator when they called with a reminder.  NOTE: This code may overlap with Facilitators to Use. |
| Usability and acceptability of receiving reminders to complete ePROs | **ePRO Reminders**  **(Opportunity; Environmental context & resources)** | Use this code when participants comment on what kind of reminders they received to complete the questionnaires and how un/helpful those reminders were.  Also use this code if participants offer suggestions for improving reminders. |
| Level of importance placed on completing ePROs and motivation for doing so/not doing so | **ePRO Importance & Motivation**  **(Motivation; Opportunity; Environmental context & resources)** | Use this code when participants discuss their motivation for completing questionnaires, how important they perceived completing the questionnaires to be, and how they fit completing questionnaires into their daily routine.  Include comments directly related to if/how ePROs would be of benefit to other patients and/or study team members (NOT personal benefits).  NOTE: Be aware that for some participants, how they fit completing the questionnaires speaks to their views on the importance and personal values/motivations (i.e., “It was easy to make time for this because I wanted my information to help others”). Be sure to use this code when responses speak to these views. |
| Facilitators to completing ePROs on a regular basis | **ePRO Facilitators to Use**  **(Opportunity; Environmental context & resources)** | Use this code when participants comment on the factors that enabled them to complete questionnaires on a regular basis.  Also use this code if participants do not provide any facilitators to completing the questionnaires (i.e., “I can’t think of anything that helped me complete the questionnaires”). |
| Barriers to completing ePROs on a regular basis | **ePRO Barriers to Use**  **(Capability; Opportunity)** | Use this code when participants comment on any barriers or concerns they experienced that impeded their ability to complete the questionnaires on a regular basis and/or delayed their completing the questionnaires.  Be sure to use this code if participants report no barriers to completing the questionnaires. |
| Benefits or personal value gained by completing ePROs | **ePRO Personal Benefits**  **(Motivation; Goals; Emotion; Reinforcement)** | Use this code when participants comment on any personal benefit or value they felt from completing the questionnaires and the extent to which they felt completing the questionnaires helped them (including prompting callbacks from care team members).  Also use this code if participants did not perceive any personal benefit or value from completing the questionnaires. |
| **Section 3. ePRO Symptom Questionnaire Response Utility** | | |
| Understanding of how clinicians will use ePRO responses and/or patient interest in reviewing responses with their care team | **Clinician Use of ePRO Responses**  **(Opportunity; Social influences; Motivation; Reinforcement)** | Use this code when participants comment on their understanding of how their clinicians plan to use or how their clinicians should use the responses they provided on the questionnaires.  Include comments about participants’ knowledge and perceptions regarding if/how clinicians may have or did use their questionnaires.  Also use this code if participants discuss their level of interest in reviewing their questionnaire responses with their care team members. |
| Level of interest in seeing their own responses and/or being able to compare their responses with other patients like them | **Patient Use of ePRO Responses**  **(Motivation; Goals)** | Use this code when participants comment on their level of interest in having access to and reviewing their own questionnaire responses, when they would like to review their responses, and their level of interest in being able to compare their responses with other patients like them. |
| Use of MyChart and acceptability of viewing ePRO responses on the portal | **MyChart Use** | Use this code when participants discuss whether they use MyChart, and if so, how they use it and what they think of being able to access their questionnaire responses on the portal. |
| **Section 4: Feasibility of ePRO Symptom Questionnaires** | | |
| Feasibility of completing ePROs at home after returning from the hospital and to what extent they needed help filling out the questionnaire | **Feasibility of Completing ePROs at Home (Capability; Knowledge; Skills; Memory/Attention; Behavioral regulation)** | Use this code when participants explicitly comment on the ease or difficulty they experienced completing the questionnaires, whether they completed the questionnaires by themselves at home or needed help, and, if they needed help at home, who helped them and how. |
| Changes or suggestions for improving the system or their own experience completing ePROs | **Changes to ePRO Delivery** | Use this code when participants offer any suggestions or changes they would like to see made to the ePRO system, to callbacks, or anything to improve their experience with ePROs. |
| **Section 5: Closing** | | |
| Whether participants recommend other patients complete ePROs | **Recommend ePROs** | Use this code when participants comment on whether they would recommend other post-op patients complete ePRO questionnaires to help improve delivery of care. |
